# Research race-specific reference values and lung function impairment, breathlessness and prognosis: Analysis of NHANES 2007–2012

**DOI:** 10.1186/s12931-022-02194-4

**Published:** 2022-10-01

**Authors:** Magnus Ekström, David Mannino

**Affiliations:** 1 Faculty of Medicine, Department of Clinical Sciences Lund, Respiratory Medicine, Allergology and Palliative Medicine, Lund University, Lund, Sweden; 2 Department of Medicine, University of Kentucky College of Medicine, Lexington, KY USA; 3 COPD Foundation, Washington, D.C USA; 4 Department of Medicine, Blekinge Hospital, SE-37185 Karlskrona, Sweden

## Abstract

**Background:**

Spirometry reference values differ by race/ethnicity, which is controversial. We evaluated the effect of race-specific references on prevalence of lung function impairment and its relation to breathlessness and mortality in the US population.

**Methods:**

Population-based analysis of the National Health and Nutrition Examination Survey (NHANES) 2007–2012. Race/ethnicity was analyzed as black, white, or other. Reference values for forced expiratory volume in one second (FEV_1_) and forced vital capacity (FVC) were calculated for each person using the Global Lung Initiative (GLI)-2012 equations for (1) white; (2) black; and (3) other/mixed people. Outcomes were prevalence of lung function impairment (< lower limit of normal [LLN]), moderate/severe impairment (< 50%pred); exertional breathlessness; and mortality until 31 December, 2015.

**Results:**

We studied 14,123 people (50% female). Compared to those for white, black reference values identified markedly fewer cases of lung function impairment (FEV_1_) both in black people (9.3% vs. 36.9%) and other non-white (1.5% vs. 9.5%); and prevalence of moderate/severe impairment was approximately halved. Outcomes by impairment differed by reference used: white (best), other/mixed (intermediate), and black (worst outcomes). Black people with FEV_1_ ≥ LLN_black_ but < LLN_white_ had 48% increased rate of breathlessness and almost doubled mortality, compared to blacks ≥ LLN_white_. White references identified people with good outcomes similarly in black and white people. Findings were similar for FEV_1_ and FVC.

**Conclusion:**

Compared to using a common reference (for white) across the population, race-specific spirometry references did not improve prediction of breathlessness and prognosis, and may misclassify lung function as normal despite worse outcomes in black people.

**Supplementary information:**

The online version contains supplementary material available at 10.1186/s12931-022-02194-4.

## Introduction

Chronic respiratory disease is the third leading cause of death worldwide [1]. Pulmonary function testing using spirometry is key to the diagnosis of chronic respiratory disease, evaluation of breathlessness, whether people qualify for interventions such as lung transplant, or can be considered to be disabled [2]. Standards exist for both the performance [3]. and interpretation [4]. of spirometry. The American Thoracic Society (ATS) recommends that ‘laboratories must select appropriate reference values for the patients being tested’ [4]. and goes on to recommend use of the Global Lung Initiative (GLI)-2012 prediction equations,[5, 6]. which establishes race–specific reference values for whites, African Americans, North East Asians, and South East Asians. Currently, race–specific reference values for lung function are the recommended standard for use internationally [6–8].

How do race–specific reference values for lung function work? The GLI-2012 prediction equations for normal lung function account for age, height, sex, and race/ethnicity [3]. While historic prediction equations would apply an ‘adjustment factor’ of 0.88 (12% less) for black populations and 0.94 (6% less) for Asian populations,[9]. the GLI-2012 equations were developed without a fixed adjustment factor but rather using race-specific populations. However, even in the GLI-2012 equations, predicted lung function levels are 10–15% lower in African Americans and South East Asians relative to whites and North East Asians [2].

Race-specific reference values are controversial. On one side of the argument is the thought that race/ethnicity is a surrogate measure that captures a number of factors predictive of poor health status and outcomes that are not really specific to a person’s racial make-up [10, 11]. The other side of the argument is that there are physiologic traits between populations that are based in genetics and captured, to some extent, by self-reported race/ethnicity [12]. Genome-wide genetic data have shown a negative correlation between the degree of African ancestry and spirometry values both in adults and children and after adjusting for socioeconomic status, healthcare access and key environmental exposures [13–15].

In other area of medicine, race-specific normal values have recently been shown to discriminate and contribute to under-diagnosing and under-treatment in socioeconomically more vulnerable groups such as Afro-Americans and are currently revised not to be specific for race/ethnicity, such as normal values for renal function [16].

We aimed to determine the effect of race-specific lung function references on prevalence of lung function impairment and its relation to breathlessness and mortality in the US population.

## Methods

### Design and population

This was a population-based analysis of the National Health and Nutrition Examination Survey (NHANES) in the US from 2007 to 2012 [17–19]. We included all people aged ≥ 18 years with data on demographics and spirometry. Data were obtained on breathlessness on exertion (available for people aged ≥ 40 years), and mortality up to 31 December 2015. The study is reported in accordance with STrengthening the Reporting of OBservational studies in Epidemiology (STROBE) guidelines [20].

### Ethics and consent to participate

Participants provided written consent to participate in NHANES using a protocol approved by the National Center for Health Statistics Research Ethics Review Board [17–19]. All the data used in the present analysis are de-identified and publicly available, and did not need additional ethical approval by the Swedish Ethical Review Authority in accordance with national research regulations.

### Assessments

Data on age, sex, and race/ethnicity were from personal interviews. Race/ethnicity was categorized as Non-Hispanic whites (whites), Non-Hispanic black (blacks), and others (Hispanic, Asian, mixed race/ethnicity, etc.). Analysis focused on comparing black vs. white, as these reference values differ the most [5]. The category other was included to reflect the entire NHANES population.

Measured weight (kg), height (cm), and spirometry were obtained using mobile examination centres. Dynamic spirometry was performed in accordance with guidelines from the ATS and European Respiratory Society (ERS) [21]. Values were recorded as the highest obtained value (pre- or post-bronchodilator). For each participant, reference values for the forced expiratory volume in one second (FEV_1_) and forced vital capacity (FVC) were calculated using the GLI-2012 equations for (1) white, (2) black, and (3) other/mixed populations [5]. Thus, for each individual we calculated three predicted reference values for FEV_1_ and FVC, respectively, for comparison.

Breathlessness on exertion was assessed using the question: ‘Have you had shortness of breath either when hurrying on the level or walking up a slight hill?’ (yes/no), which corresponds to a breathlessness level of ≥ 1 point on the modified Medical Research Council (mMRC) scale [22]. Breathlessness data were available in NHANES for people 40 years or older. Mortality was assessed using standardized NHANES procedures up to 31 December 2015.

### Statistical analyses

The study population was weighted (using published NHANES weights for people undergoing examinations including spirometry), to represent the non-institutionalized US population during the six year period. For all analyses, variance estimates were produced using Taylor Series Linearization methods,[23]. as recommended for NHANES.

Data were tabulated and compared between race/ethnicity groups using means (standard deviation [SD]) for normally distributed continuous variables, and frequency (percentage) for categorical variables. Lung function was evaluated as FEV_1_ in the main analyses. Similar analyses of FVC and FEV_1_/FVC are reported in the supplement.

Outcomes were compared between race/ethnicity groups (white, black, and other) in terms of: (1) predicted normal values using each race-specific prediction equation (white, black, or other/mixed); (2) prevalence of impaired lung function, defined as value < the lower limit of normal (LLN, set at a z-score of -1.645) using each race-specific prediction equation;[6]. and the prevalence of moderate to severe impairment, defined as < 50% of the predicted normal in accordance with GOLD (Global Initiative for Obstructive Lung Disease);[7]. (3) prevalence of breathlessness; and (4) mortality. As each prediction equation was applied to the same people, the effects of applying different race–specific reference values were independent of (adjusted for) participant characteristics by design.

Breathlessness and mortality were compared by race/ethnicity and lung function impairment (defined using different race-specific prediction equations) using five mutually exclusive categories: ‘White Normal’ (white race/ethnicity, value ≥ LLN_white_); ‘White Abnormal’ (white race/ethnicity, value < LLN_white_); ‘Black Normal’ (white race/ethnicity, value ≥ LLN_white_); ‘Black Abnormal (White Standard)’ (black race/ethnicity, value < LLN_white_ but ≥ LLN_black_); or ‘Black Abnormal (Black Standard)’ (black race/ethnicity and value < LLN_white_ and < LLN_black_). As normal values for FEV_1_ and FVC were higher for all persons using white than black prediction equations, all values < LLN_black_ were also < LLN_white_. Probability of breathlessness was analyzed using multinomial logistic regression and was expressed as relative rate ratios (RRR). Mortality was analyzed using Cox proportional-hazards regression and expressed as hazard ratios (HR). Outcome analyses were not performed for FEV_1_/FVC as predicted values were similar using the difference race-specific equations.

Associations with breathlessness and mortality were also analyzed for lung function impairment using each race–specific prediction equation in the whole population, adjusting for age, sex and body mass index (BMI). The accuracy of predicting breathlessness and mortality was compared, between using race-specific vs. white reference values, by the models’ percentage correctly classified and C-statistic, respectively.

Estimates were reported with 95% confidence intervals (CIs). Statistical analyses were performed with Stata version 16.0 (StataCorp LP; College Station, TX).

## Results

A total 14,123 people (50% female) were studied with race/ethnicity self-reported as white (n = 5,928), black (n = 3,130), or other (n = 5,065). Compared with the two other (non-white) groups, white people were slightly older and had somewhat higher absolute FEV_1_ and FVC values, with more similar FEV_1_/FVC ratio, whereas the sex distribution and BMI was similar between race/ethnicity groups (Table 1). Breathlessness was more prevalent among black people, as compared with white and other (Table 1; p < 0.001 for both comparisons).

**Table 1:** Characteristics and lung function by race/ethnicity

Factor	White people	Black people	Other
N	5,928	3,130	5,065
Age, mean (SD)	45.7 (16.0)	42.1 (15.8)	39.5 (14.5)
Female, %	50.3%	53.7%	48.3%
Weight (kg), mean (SD)	83.0 (20.7)	87.9 (24.0)	76.7 (19.2)
Height (cm), mean (SD)	170.7 (9.8)	169.6 (9.5)	165.1 (9.6)
Body mass index (kg/m^2^), mean (SD)	28.4 (6.5)	30.6 (8.1)	28.1 (6.2)
FEV_1_ (L), mean (SD)	3.3 (0.9)	2.9 (0.8)	3.2 (0.8)
FVC (L), mean (SD)	4.3 (1.1)	3.6 (1.0)	4.0 (1.0)
FEV_1_/FVC, mean (SD)	0.77 (0.08)	0.80 (0.08)	0.81 (0.07)
Breathlessness prevalence*, %	29.3%	33.7%	24.4%
Deaths by December 31, 2015, %	3.0%	3.5%	2.2%

Characteristics are weighted to represent the mean US population in National Health and Nutrition Examination Survey (NHANES) 2007–2012. * Breathlessness prevalence is in people aged 40 years or older. Abbreviations: FEV_1_ = forced expiratory volume in one second. FVC = forced vital capacity

### Prevalence of lung function impairment

The predicted normal FEV_1_ was highest using the equation for whites, intermediate using that for other/mixed, and lowest when using the equation for black people (Table 2). This pattern was seen in all race/ethnicity groups. In black people the predicted normal FEV_1_ dropped from 3.5 L using the white equation to 3.0 L using the black equation, a decrease by 14%.

**Table 2:** Prevalence of impaired FEV_1_ by race/ethnicity and race-specific reference value used

Factor	White people	Black people	Other
N	5,928	3,130	5,065
**Predicted normal FEV**_**1**_, **mean (SD)**			
White reference values	3.5 (0.81)	3.5 (0.79)	3.4 (0.75)
Other/mixed reference values	3.2 (0.76)	3.2 (0.73)	3.1 (0.70)
Black reference values	3.0 (0.68)	3.0 (0.66)	2.9 (0.63)
**Prevalence of impaired FEV**_**1**_ **(< LLN) using, %**			
White reference values	8.5%	36.9%	9.5%
Other/mixed reference values	5.1%	21.3%	4.4%
Black reference values	2.4%	9.3%	1.5%
**Prevalence of moderate/severe FEV**_**1**_ **impairment (< 50%pred) using, %**			
White reference values	0.8%	1.7%	0.5%
Other/mixed reference values	0.5%	1.1%	0.3%
Black reference values	0.4%	0.8%	0.2%

Reference values by GLI-2012 [5]. For abbreviations, see Table 1

The choice of race/ethnicity references strongly influenced the prevalence of lung function impairment (Table 2). Compared with the equation for white people, black reference values identified markedly fewer cases of impaired FEV_1_, both among whites (2.4% vs. 8.5%), and especially in black people (9.3% vs. 36.9%), as well as in other races (1.5% vs. 9.5%). Overall, compared with the reference values for blacks, white reference values identified about four times as many people as having impaired lung function, and identification of moderate to severe impairment (< 50% predicted) was approximately doubled (Table 2). Similar findings were seen when analyzing FVC instead of FEV_1_ (supplemental Table S1). In contrast, the predicted FEV_1_/FVC and the prevalence of a reduced ratio (< LLN) were similar across the different race-specific equations (supplemental Table S2).

### Lung function and outcomes

People with impaired lung function (FEV_1_ < LLN compared with those with ≥ LLN) had increased rates of breathlessness and mortality across the whole population, but the magnitude of increase differed by the race-specific reference value used: <LLN_black_ (worst outcomes), <LLN_other/mixed_ (intermediate outcomes), and < LLN_white_ (best outcomes). The associations between impaired lung function and breathlessness were, for each race–specific reference value: LLN_black_ (RRR 4.6; 95% CI, 3.2–6.6), LLN_other/mixed_ (RRR 3.4; 95% CI, 2.7–4.4), and LLN_white_ (RRR 2.8; 95% CI, 2.4–3.3). Corresponding estimates for mortality were: for LLN_black_ (HR 3.5; 95% CI, 2.4–5.2), LLN_other/mixed_ (HR 2.8; 95% CI, 2.1–3.6), and LLN_white_ (HR 2.6; 95% CI, 2.1–3.4).

**Fig. 1:**
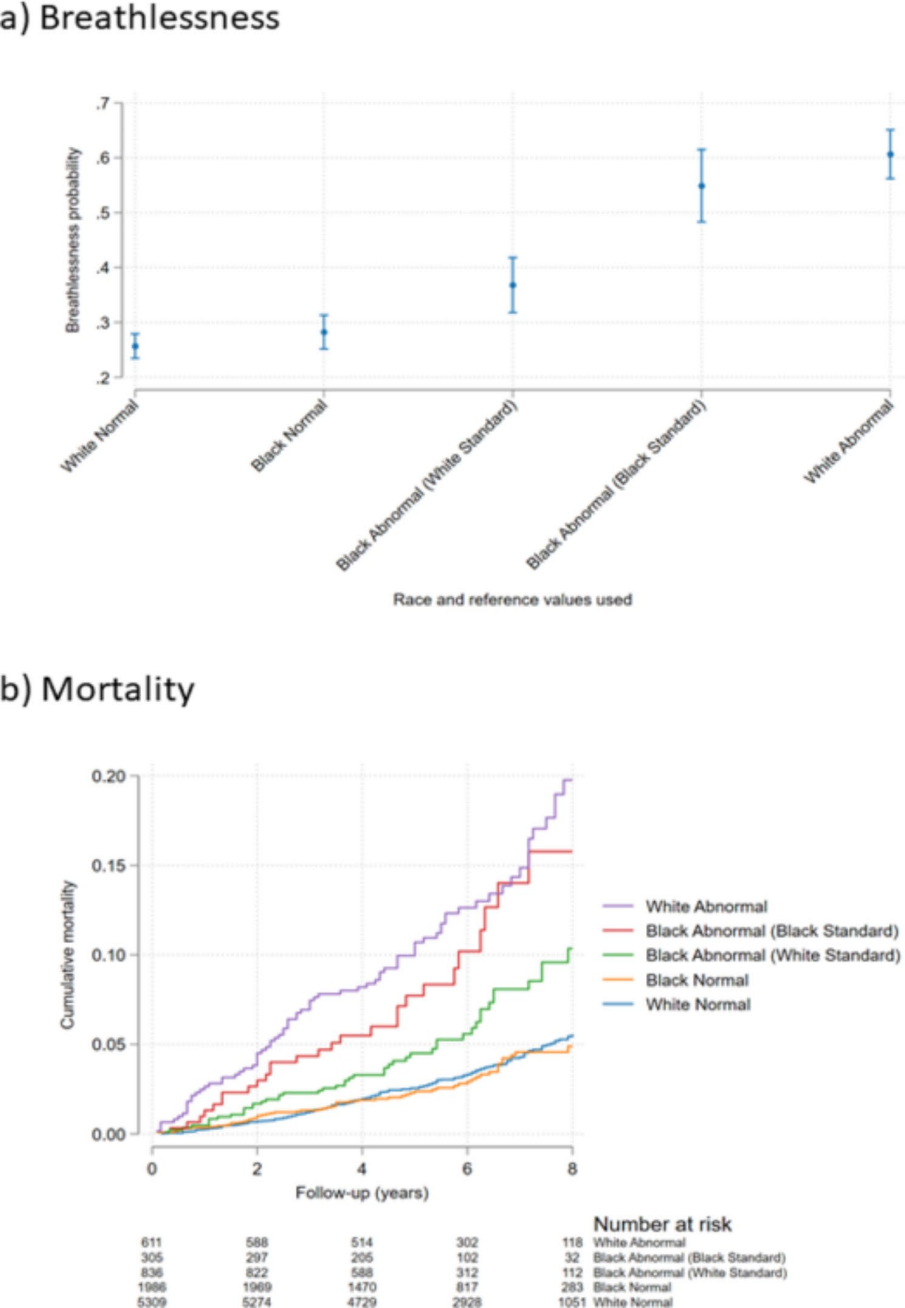
Outcomes by race/ethnicity and FEV_**1**_ impairment defined using white and/or black normal values, in terms of (a) breathlessness, and (b) mortality. Breathlessness probability was analyzed using multinomial logistic regression, and mortality using Cox proportional hazards regression. Impaired lung function was defined as a forced expiratory volume in one second (FEV_1_) < lower limit of normal (LLN) using GLI-2012 predicted normal values for white and black people, respectively [5]. Groups were categorized by race/ethnicity and FEV_1_ impairment according to different race-specific prediction equations as: ‘White Normal’ (white race/ethnicity with FEV_1_ ≥ LLN_white_); ‘Black Normal’ (black race/ethnicity with FEV_1_ ≥ LLN_white_); ‘Black Abnormal (White Reference)’ (black race/ethnicity with FEV_1_ < LLN_white_ but ≥ LLN_black_); ‘Black Abnormal (Black Reference)’ (black race/ethnicity and FEV_1_ < LLN_white_ and < LLN_black_); and ‘White Abnormal’ (white race/ethnicity and FEV_1_ < LLN_white_). The main finding is that black people who were categorized as having a normal FEV_1_ using LLN_black_ but not using LLN_white_ had increased breathlessness prevalence and mortality compared with people categorized as normal using reference values for white. Thus, black reference values misclassify black people as having normal lung function despite having worse outcomes. When defining normality using LLN_white_ for all, people with normal FEV_1_ had similar breathlessness and mortality in both white and black people

Outcomes by race/ethnicity (black or white) and level of FEV_1_ impairment defined using the different race–specific reference values are shown in Fig. 1. Black people who were categorized as having a normal FEV_1_ using LLN_black_ but not using LLN_white_ had increased breathlessness prevalence (RRR 1.48; 95% CI, 1.13–1.94) and mortality (HR 1.87; 95% CI, 1.42–2.46) compared with people categorized as normal using references for white people. Thus, black reference values classified these black people as having normal lung function despite having worse outcomes. When defining normality using LLN_white_ for all, people with normal FEV_1_ had similarly low rates of breathlessness and mortality in both white and black people.

These findings were confirmed in Cox regression adjusted for age, sex and BMI (supplemental Table S3). Prediction of outcomes (adjusted for the same factors) was not improved by using race-specific references compared to using the same reference equation (white) across the population, neither for breathlessness (71% correctly classified by both models) or mortality (C-statistic 0.77 for both models).

All findings were similar when analyzing FVC instead of FEV_1_ (Table S4 and Figure S1 in the supplement).

## Discussion

In this population-based study across the US population, using race-specific reference values (GLI-2012)[5]. results in markedly lower identified prevalence of lung function impairment (FEV_1_ or FVC) in non-white people, including of moderate to severe impairment. In black people, race-specific (black) reference values identified only ¼ of cases (9.3% vs. 36.9%) of impaired FEV_1_ as compared with white reference values – and those identified by the black references had significantly worse lung function, more breathlessness and increased mortality. Importantly, race-specific references classified black people as having normal lung function despite having substantially increased rates of breathlessness and mortality. Black people with normal lung function according to black but impaired according to white references (FEV_1_ ≥ LLN_black_ but < LLN_white_) had 48% increased rate of breathlessness and almost doubled mortality, as compared with black people with normal FEV_1_ by white standards. People with normal lung function according to white reference values had similar good prognosis across all the race/ethnicity groups. Using race-specific references did not predict the outcomes better than using white references across the population.

These findings have important implications for evaluation of lung function and respiratory disease. Firstly, while race-specific reference values, which are currently endorsed by major international guidelines,[6–8]. are useful for categorizing airflow obstruction (based on FEV_1_/FVC ratio), race-specific references may misclassify and underdiagnose reduced lung function in non-white people, including moderate to severe impairment. As we show, race-specific references (compared to white references) classified lung function as normal despite worse outcomes in as many as 28% of black people, corresponding to 13.1 million people in the US alone [24]. A particularly alarming finding was that half of cases of moderate to severe lung function impairment were not detected – which could lead to insufficient treatment or delayed interventions such as evaluation for lung transplantation. Thus, using lung function references that are specific for each race/ethnicity may contribute to under diagnosis of lung function impairment and disability, failure to identify impaired lung function as a contributing cause in evaluation of breathlessness, misclassify the association between lung function and outcomes, and potentially lead to insufficient or delayed treatment and compounded race-related health inequities in the community. Second, these findings suggest that lung function should be assessed using a common prediction equation that is not specific for race/ethnicity across the population. The choice of which common prediction equation to use may vary depending on aim of the assessment and cannot be inferred from the present analysis. In research and clinical medicine, the aims of spirometry are mainly to evaluate whether lung function is normal or impaired, whether breathlessness is related to impaired lung function, evaluate the severity of respiratory disease needing treatment or further evaluation, and to predict prognosis. While defining the optimal reference equation to use lies beyond the scope of this analysis, we found white references to be more sensitive than the GLI-2012 mixed/other reference values, which were previously proposed for use across mixed populations [5]. White references were more sensitive to identify reduced lung function that was still associated with substantial morbidity in terms of increased breathlessness and mortality rates. This was seen across all the race/ethnicity groups. Thus, white reference values identified lung function reductions that were smaller or earlier, which should be appropriately evaluated and may be more amenable to treatment. Which reference equations that should be used across the populations need further validation.

The present findings extend previous reports that race-related differences in mortality were attenuated by applying using the same prediction equation (reference values for whites) across the population [25]. The findings are consistent with recent reports that using race-specific reference values did not improve prediction of respiratory morbidity or mortality in a large cohort study[26], and even predicted clinically important outcomes worse (than using a common reference) in black and white people at high risk of COPD (n = 2,652) [27]. A study using NHANES III data found that the lower lung function in black people had similar implications for all-cause mortality as similarly low lung function in white people,[28]. and that multiracial reference equations accounting for age, sex and income showed yielded similar associations between lung function and mortality in black and white people. Taken together, these previous data support our findings that using race-specific references may widen inequities in health.

An important consideration is, however, that using white (and not race-specific) references resulted in a markedly higher prevalence of lung function impairment among non-white people. Adopting the GLI white reference for black people could result in almost 40% of African American adults being categorized as having reduced lung function, with potentially significant consequences in terms of overdiagnosis, overtreatment, exclusion of black people with lung function on the lower side of normal from accessing medical/surgical treatment and jobs that require lung function thresholds. It is likely that in many of those people, the lower lung function may not reflect underlying respiratory disease but the influence of other adverse factors and exposures. To avoid over diagnosis, this highlights the importance of spirometry being interpreted as part of a broader evaluation and clinical context. Earlier detection of lung function impairment, associated with other symptoms and limitations, may facilitate identification of adverse early life exposure, life style and environmental factors that may be amenable to interventions to improve health.

Our findings of lower lung function and worse outcomes in black people are consistent with data that Afro-Americans have more undiagnosed obstructive lung disease, health care contacts and hospitalizations,[29]. [11]. and are less likely to be listed for lung transplantation [30]. Airflow obstruction and reduced lung function strongly associates with lower socioeconomic status and poverty at individual and community levels across multiple countries, independent of factors such as age, sex, and exposure to smoking and tuberculosis [31]. In the recent study by Baugh et al., controlling for comorbid disease and measures of adversity weakened the association between race/ethnicity and FEV_1_, suggesting that differences in lung function related to race/ethnicity at least partly reflect different life circumstances and exposures [27]. It is increasingly acknowledged that race/ethnicity is to a large extent a social construct [2, 11]. Genetic and environmental factors inseparably interact in multiple and complex ways to influence all aspects of life including lung function, through prenatal and early life factors, circumstances throughout life, and over the generations [2]. In the Eight America’s project, Murray et al. described large disparities in mortality across race-county groupings and concluded that these differences could not be explained by race/ethnicity, income, or basic health-care access and utilization alone [32]. As pointed out,[2]. the lower lung function in disadvantaged groups including Afro-Americans might, to an extent, reflect a higher accumulated exposure to adverse exposures and not disease. But as we show, using race-specific lung function references may obscure the higher prevalence of impairment in these populations, misclassify black people as healthy despite having worse outcomes, and contribute to under diagnosis of disease or presence of modifiable health exposures that could, when appreciated, be modified [2]. An example of the influence of environmental factors from another field is the generational change in health outcomes when comparing the population of southern Europe to northern Europe [33]. Even though these populations had similar race/ethnicity distributions, large differences in both adult height and childhood mortality seen in 1950 had largely disappeared by 1980 [33].

The suggestion that reference values for lung function should not be specific by race/ethnicity is consistent with similar developments in other medical areas, including for tests in haematology, and revised, non-race-specific, reference values for kidney function [16, 34, 35].

Strengths of the present study include the use of a well characterized, large database representative for the racially diverse non-institutionalized US population. Race-specific prediction equations for normal lung function (FEV_1_ and FVC) were evaluated using the international GLI-2012 reference values developed to be applicable globally, in accordance with guidelines [5, 7, 8]. By comparing the predictions in the same population, the analyses were independent of differences in participant characteristics. Reference values were evaluated against clinically important outcomes in terms of prevalence of impairment, breathlessness and mortality. A limitation of the present study is that data pertain to the US population, and studies in other settings are needed.

In conclusion, compared to using a common reference (for white) across the population, race-specific spirometry references did not improve prediction of breathlessness and prognosis, and may misclassify lung function as normal despite worse outcomes in black people. This race-related bias can be attenuated by applying a similar reference across the population, and which common references to use calls for further research.

## Electronic supplementary material

Below is the link to the electronic supplementary material.

Supplementary Material 1

## Data Availability

All data used are anonymized and publicly available. Analysis scripts (for Stata) will be made available from the authors upon reasonable request.
