# Correction: Race-specific reference values and lung function impairment, breathlessness and prognosis: analysis of NHANES 2007–2012

**DOI:** 10.1186/s12931-023-02351-3

**Published:** 2023-02-03

**Authors:** Magnus Ekström, David Mannino

**Affiliations:** 1 Faculty of Medicine, Department of Clinical Sciences Lund, Respiratory Medicine, Allergology and Palliative Medicine, Lund University, Lund, Sweden; 2 Department of Medicine, University of Kentucky College of Medicine, Lexington, KY USA; 3 COPD Foundation, Washington, DC USA; 4 Department of Medicine, Blekinge Hospital, 37185 Karlskrona, Sweden

**Correction: Respiratory Research (2022) 23:271** 10.1186/s12931-022-02194-4

Following the publication of the original article [1], the authors identified an error in the article title. That is, the article type “Research” was erroneously added to the title. It has been corrected in this correction and the original article has been updated.

Correct title is: Race-specific reference values and lung function impairment, breathlessness and prognosis: analysis of NHANES 2007–2012.
