# Supplementary material for: Research race-specific reference values and lung function impairment, breathlessness and prognosis: Analysis of NHANES 2007–2012
Source: Respir Res. 2022 Oct 1;23:271. doi: 10.1186/s12931-022-02194-4 (PMC9526909; doi:10.1186/s12931-022-02194-4)
Supplement: Supplementary file 1 — Supplementary Material 1 [file 12931_2022_2194_MOESM1_ESM.pdf]

Supplementary material

Race-Specific Reference Values and Lung Function Impairment, Breathlessness and Prognosis: analysis of NHANES 2007-2012

Table S1. Prevalence of impaired FVC by race/ethnicity and reference values used

Factor	White people	Black people	Other
N	5,928	3,130	5,065
**Predicted normal FVC, mean (SD)**			
White reference values	4.28 (0.98)	4.26 (0.94)	4.10 (0.89)
Other/mixed reference values	3.94 (0.90)	3.93 (0.87)	3.78 (0.82)
Black reference values	3.63 (0.81)	3.62 (0.78)	3.48 (0.74)
**Prevalence of impaired FVC (<LLN) using, %**			
White reference values	4.7%	32.5%	8.5%
Other/mixed reference values	2.1%	19.2%	3.5%
Black reference values	0.7%	7.0%	1.5%
**Prevalence of moderate/severe FVC impairment (<50%pred) using, %**			
White reference values	0.1%	0.7%	0.2%
Other/mixed reference values	0.1%	0.4%)	0.1%
Black reference values	<1%	0.2%	<1%

Reference values by GLI-2012.[5] *Abbreviations:* FVC = forced vital capacity; LLN = lower limit of normal; pred = predicted normal value.

Table S2. Prevalence of impaired FEV_1/FVC by race/ethnicity and reference values used

Factor	White people	Black people	Other
N	5,928	3,130	5,065
**Predicted normal FEV_1/FVC, mean (SD)**			
White reference values	0.81 (0.03)	0.82 (0.03)	0.83 (0.03)
Other/mixed reference values	0.82 (0.03)	0.83 (0.03)	0.83 (0.03)
Black reference values	0.82 (0.03)	0.82 (0.03)	0.83 (0.03)
**Prevalence of impaired FEV_1/FVC (<LLN) using, %**			
White reference values	9.3%	6.6%	4.0%
Other/mixed reference values	13.7%	9.7%	6.2%
Black reference values	11.1%	7.9%	4.9%
**Prevalence of FEV_1/FVC < 0.7, %**	13.1%	8.2%	4.8%

Reference values by GLI-2012.[5] *Abbreviations:* FEV_1 = forced expired volume in one second; FVC = forced vital capacity; LLN = lower limit of normal; pred = predicted normal value.

Table S3. Adjusted associations with breathlessness and mortality by race/ethnicity and FEV_1 impairment

	Breathlessness RRR (95% CI)		Mortality Hazard ratio (95% CI)	
**Group**	**Crude**	**Adjusted***	**Crude**	**Adjusted***
White Normal	1 (ref)	1 (ref)	1 (ref)	1 (ref)
Black Normal	1.14 (0.95–1.37)	1.00 (0.84–1.20)	1.10 (0.83–1.47)	1.36 (1.01–1.83)
Black Abnormal (White Standard)	1.69 (1.36–2.08)	1.55 (1.24–1.94)	2.07 (1.51–2.83)	2.64 (1.88–3.70)
Black Abnormal (Black Standard)	3.52 (2.63–4.71)	2.91 (2.15–3.94)	3.46 (2.30–5.19)	3.27 (2.16–4.95)
White Abnormal	4.46 (3.64–5.46)	4.27 (3.45–5.30)	4.05 (2.84–5.78)	3.08 (2.16–4.38)

Breathlessness data were available and analyzed in people aged 40 years or older. Groups are categorized similar to in Figure 1. *Adjusted for age, sex, and body mass index. Abbreviations: CI = confidence interval; FEV_1 = forced expired volume in one second; RRR = relative rate ratio.

Table S4. Adjusted associations with breathlessness and mortality by race/ethnicity and FVC impairment

	Breathlessness RRR (95% CI)		Mortality Hazard ratio (95% CI)	
**Group**	**Crude**	**Adjusted***	**Crude**	**Adjusted***
White Normal	1 (ref)	1 (ref)	1 (ref)	1 (ref)
Black Normal	1.10 (0.93–1.30)	1.01 (0.85–1.19)	1.04 (0.78–1.40)	1.30 (0.97–1.74)
Black Abnormal (White Standard)	1.71 (1.37–2.13)	1.50 (1.20–1.87)	2.00 (1.39–2.89)	2.65 (1.79–3.91)
Black Abnormal (Black Standard)	2.42 (1.77–3.31)	1.76 (1.27–2.43)	3.48 (2.28–5.32)	3.63 (2.29–5.75)
White Abnormal	4.02 (2.94–5.49)	3.12 (2.18–4.44)	4.22 (2.80–6.36)	3.43 (2.36–4.99)

Breathlessness data were available and analyzed in people aged 40 years or older. Groups are categorized similar to in Figure S1. *Adjusted for age, sex, and body mass index. Abbreviations: CI = confidence interval; FVC = forced vital capacity; RRR = relative rate ratio.

Figure S1. Outcomes by race/ethnicity and FVC impairment defined using reference values for white and/or black people, in terms of a) breathlessness, and b) mortality. Breathlessness probability was analyzed using logistic regression, and mortality using Cox proportional hazards regression. Impaired lung function was defined as a forced vital capacity (FVC) < lower limit of normal (LLN) using GLI-2012 predicted normal values for white and black people, respectively.[5] Groups were categorized by race/ethnicity and FVC impairment according to different race-specific prediction equations as: 'White Normal' (white race/ethnicity with FVC ≥ LLN_{white}); 'Black Normal' (black race/ethnicity with FVC ≥ LLN_{white}); 'Black Abnormal (White Reference)' (black race/ethnicity with FVC < LLN_{white} but ≥ LLN_{black}); 'Black Abnormal (Black Reference)' (black race/ethnicity and FVC < LLN_{white} and <LLN_{black}); and 'White Abnormal' (white race/ethnicity and FVC < LLN_{white}). The main finding is that black people who were categorized as having a normal FVC using LLN_{black} but not using LLN_{white} had increased breathlessness prevalence and mortality compared with people categorized as normal using reference values for white. Thus, black reference values misclassify black people as having normal lung function despite having worse outcomes. When defining normality using LLN_{white} for all, people with normal FVC had similar breathlessness and mortality in both white and black people.

a) Breathlessness

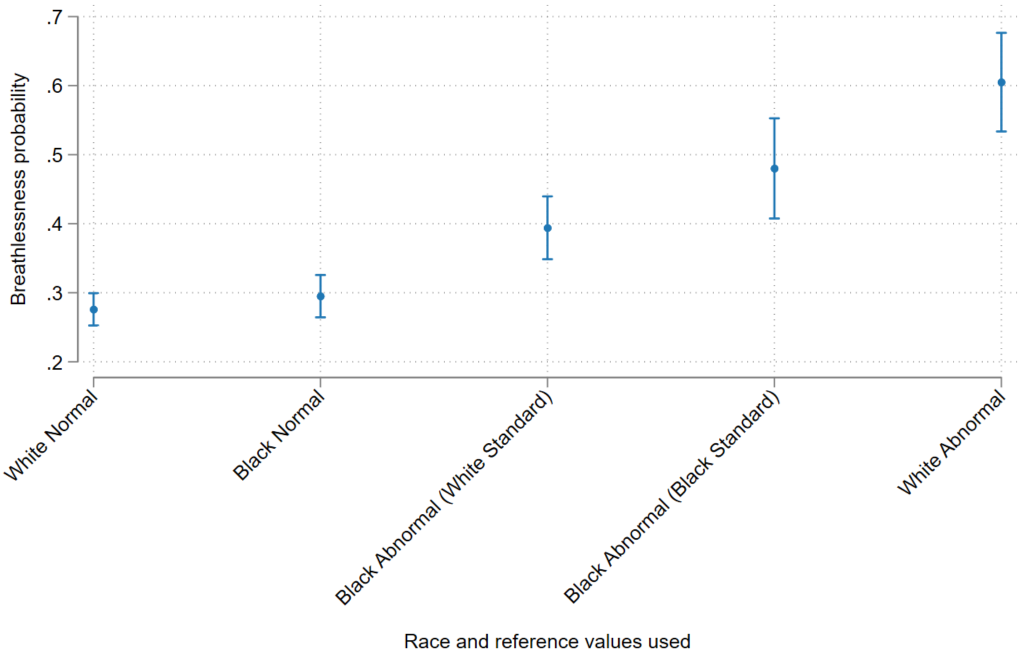

b) Mortality

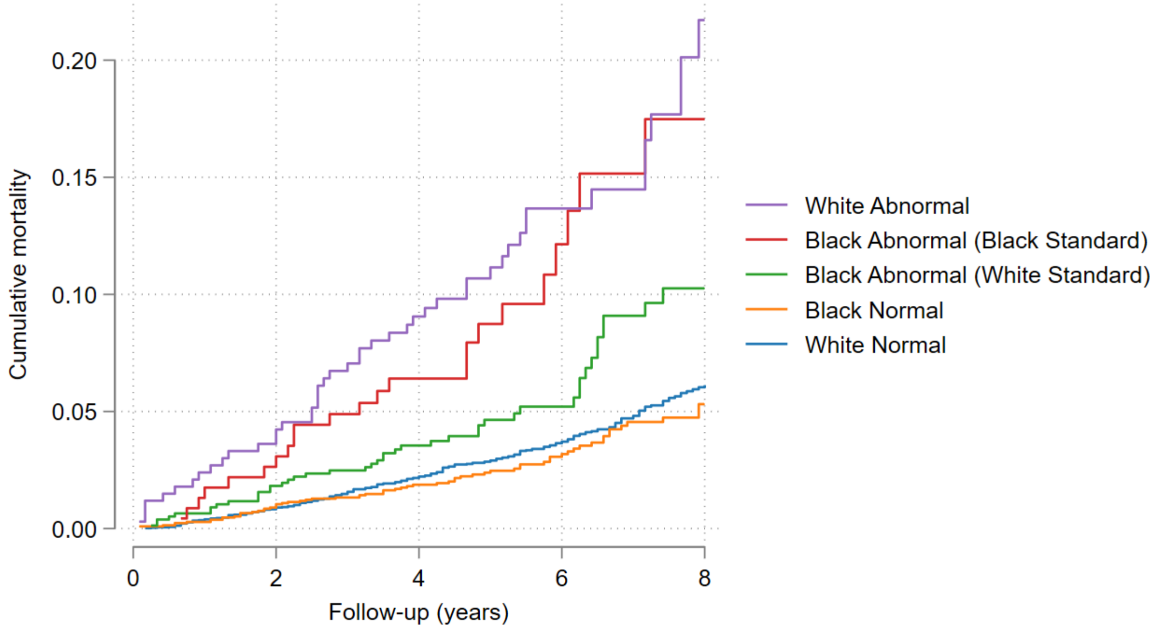

Number at risk

337	325	282	153	60	White Abnormal
230	224	152	73	21	Black Abnormal (Black Standard)
774	760	547	275	91	Black Abnormal (White Standard)
2123	2104	1564	883	315	Black Normal
5583	5537	4961	3077	1109	White Normal
